# Establishment of a corneal ulcer prognostic model based on machine learning

**DOI:** 10.1038/s41598-024-66608-7

**Published:** 2024-07-12

**Authors:** Meng-Tong Wang, You-Ran Cai, Vlon Jang, Hong-Jian Meng, Ling-Bo Sun, Li-Min Deng, Yu-Wen Liu, Wen-Jin Zou

**Affiliations:** 1 Department of Ophthalmology, The First Affiliated Hospital of Guangxi Medical University, 22 Shuangyong Road, Nanning, Guangxi Zhuang Autonomous Region China; 2 Qi Dian Fu Liu Technology Co.Ltd, Beijing, China; 3 Department of Ophthalmology, The First Affiliated Hospital of Guangxi University of Chinese Medicine, Nanning, China; 4 Department of Ophthalmology, Ruikang Hospital Affiliated to Guangxi University of Chinese Medicine, Nanning, China; 5 Department of Ophthalmology, Guangxi Zhuang Autonomous Region People’s Hospital, Nanning, China; 6 School of Medicine, Eye Institute of Xiamen University, Xiamen University, Xiamen, Fujian China

**Keywords:** Artificial intelligence, Deep-learning algorithm, Machine learning, Cornea, Corneal ulcer, Diseases, Medical research, Risk factors

## Abstract

Corneal infection is a major public health concern worldwide and the most common cause of unilateral corneal blindness. Toxic effects of different microorganisms, such as bacteria and fungi, worsen keratitis leading to corneal perforation even with optimal drug treatment. The cornea forms the main refractive surface of the eye. Diseases affecting the cornea can cause severe visual impairment. Therefore, it is crucial to analyze the risk of corneal perforation and visual impairment in corneal ulcer patients for making early treatment strategies. The modeling of a fully automated prognostic model system was performed in two parts. In the first part, the dataset contained 4973 slit lamp images of corneal ulcer patients in three centers. A deep learning model was developed and tested for segmenting and classifying five lesions (corneal ulcer, corneal scar, hypopyon, corneal descementocele, and corneal neovascularization) in the eyes of corneal ulcer patients. Further, hierarchical quantification was carried out based on policy rules. In the second part, the dataset included clinical data (name, gender, age, best corrected visual acuity, and type of corneal ulcer) of 240 patients with corneal ulcers and respective 1010 slit lamp images under two light sources (natural light and cobalt blue light). The slit lamp images were then quantified hierarchically according to the policy rules developed in the first part of the modeling. Combining the above clinical data, the features were used to build the final prognostic model system for corneal ulcer perforation outcome and visual impairment using machine learning algorithms such as XGBoost, LightGBM. The ROC curve area (AUC value) evaluated the model’s performance. For segmentation of the five lesions, the accuracy rates of hypopyon, descemetocele, corneal ulcer under blue light, and corneal neovascularization were 96.86, 91.64, 90.51, and 93.97, respectively. For the corneal scar lesion classification, the accuracy rate of the final model was 69.76. The XGBoost model performed the best in predicting the 1-month prognosis of patients, with an AUC of 0.81 (95% CI 0.63–1.00) for ulcer perforation and an AUC of 0.77 (95% CI 0.63–0.91) for visual impairment. In predicting the 3-month prognosis of patients, the XGBoost model received the best AUC of 0.97 (95% CI 0.92–1.00) for ulcer perforation, while the LightGBM model achieved the best performance with an AUC of 0.98 (95% CI 0.94–1.00) for visual impairment.

## Introduction

Corneal ulcer is a serious corneal disease and leading cause of blindness worldwide^1–3^. Corneal ulcers often lead to scarring and astigmatism, and significant loss of vision is a common consequence. In severe cases, perforation, scleral involvement, and endophthalmitis may occur^4^. The analysis of corneal ulcer severity during clinical examination is highly subjective and observer-dependent^5^. An ophthalmologist mainly determines the prognosis of the disease based on the severity of ocular lesions, such as corneal ulcers, corneal scars, corneal neovascularization, anterior corneal abscess, and corneal descementocele^6–9^. Due to the close correlation between lesion features and clinical prognosis, ophthalmologists highly consider these lesions and usually evaluate them when making treatment decisions to reduce morbidity and further consequences such as corneal perforation^10,11^.

Advances in machine learning and computer vision have provided new ways to improve medicine, particularly in pattern recognition and image classification. Deep-learning algorithms have been applied to the segmentation and classification of various medical images^12^. In ophthalmology, most current AI algorithms are deep learning algorithms developed based on various imaging modes^13–15^. In the ocular surface area, algorithms have been developed to detect and distinguish types of microbial corneal ulcer and to quantify its characteristics^16^. However, to our knowledge, no deep learning-based prognostic model has been established for patients with corneal ulcer.

In this study, we used multi-centre data sets to develop and test an automatic prognostic model system that could segment, classify, and accurately quantify four corneal ulcer lesions on slit-lamp images and predict the perforation outcome combined with clinical data. The aim is to assist clinicians in accurately quantifying and visualising corneal ulcer lesions, as well as predicting corneal ulcer perforation and visual impairment.

## Methods

### Study participants

**Figure 1:**
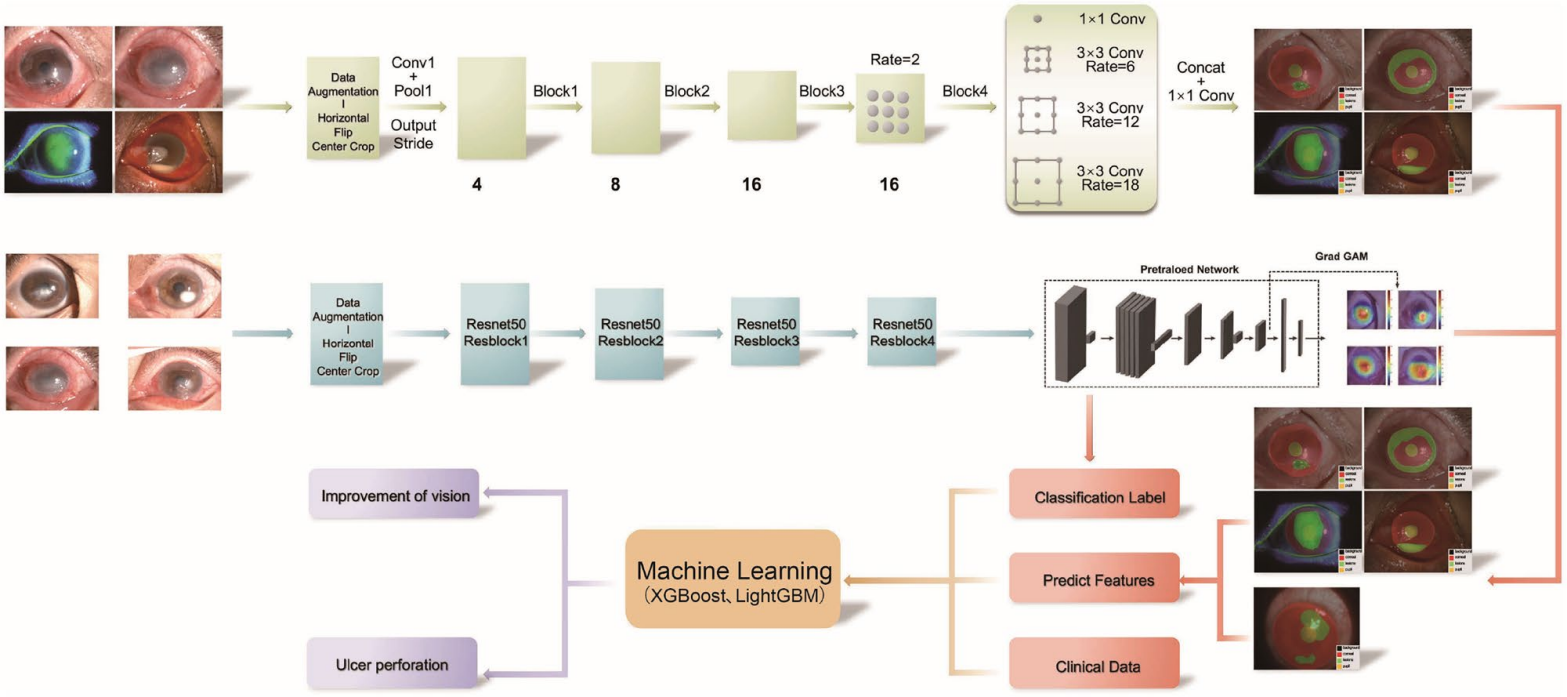
This is the workflow of the study.

In the first part of the modelling, 4973 slit-lamp images of patients with corneal ulcer were retrospectively collected from the First Affiliated Hospital of Guangxi Medical University, the Ophthalmic Research Center of Xiamen University, and the Ophthalmic Research Centre of Sun Yat-Sen University (SUSTEC-SYSU database)^17^ from 2017 to 2019. The images were divided into five datasets by four ocular surface doctors depending on the lesion characteristics: Corneal ulcers (1960 images), corneal scars (1734 images), corneal neovascularization (947 images), anterior corneal abscess (234 images), and corneal descementocele (98 images). Each data set is divided into training set, test set and verification set according to different central sources. For each image, the region of interest (ROI) was manually annotated using LabelMe (4.5.6) software. Pathological ROIs included corneal scarring, neovascularization, anterior corneal abscess, and descementocele on the diffuse white-light slit-lamp image. Corneal ulcer lesions were annotated using a diffuse blue light slit-lamp image. The lesions were labelled by a keratopathy expert and three ophthalmologists. Non-pathological ROIs included limbus and pupil markings and were identified by three ophthalmologists. Each slit-lamp image contained at least one lesion as well as limbus and pupil markings. In the second part of the modelling, clinical data and 1010 slit-lamp images were retrospectively collected from 240 corneal ulcer patients admitted between December 2019 and May 2022. The data was randomly partitioned into a training set and a validation set in an 8:2 ratio. The primary inclusion criteria were patients with etiologically confirmed infectious corneal ulcers. The primary exclusion criteria were patients who underwent surgical treatment during diagnosis and treatment. (Supplemental Fig. 1) For eligible patients, the following demographic and clinical data were collected: Age of onset, best-corrected vision of the affected eye, type of corneal ulcer, and slit-lamp images from two light sources. Written or verbal informed consent was not obtained from any participant, as this study had a non-interventional retrospective design and all data were analysed anonymously (Fig. 1).

### Data preprocessing and augmentation

For the segmentation model, the shortest side of all the data was randomly resized to 260–1040, and the long side was changed in equal proportions. Subsequently, 480 × 480 random clipping was performed. In training, considering that the dataset was generally positive, vertical flipping was not applied, and considering the symmetry of the left and right eyes, a 50% probability of horizontal flipping was introduced to expand the scale of our training dataset.

For the classification model, the data were randomly cropped to 224 × 224 pixels, which was adapted to the ResNSet50 model and transfer learning. Similar to the segmentation model, only horizontal flipping was introduced as a data augmentation technology.

### Segmentation model

An automatic ROI segmentation model of the slit-lamp image was performed for five different tasks: Corneal descementocele, corneal scar, corneal neovascularization, blue-light corneal ulcer, and anterior corneal abscess. Common deep-learning segmentation models, such as FCN, U-net, and DeepLabV3, have been investigated. Combined with our own application scenarios and the performance of the models in the benchmark, the DeeplabV3 model was selected as the basis for all the subsequent models.

**Figure 2:**
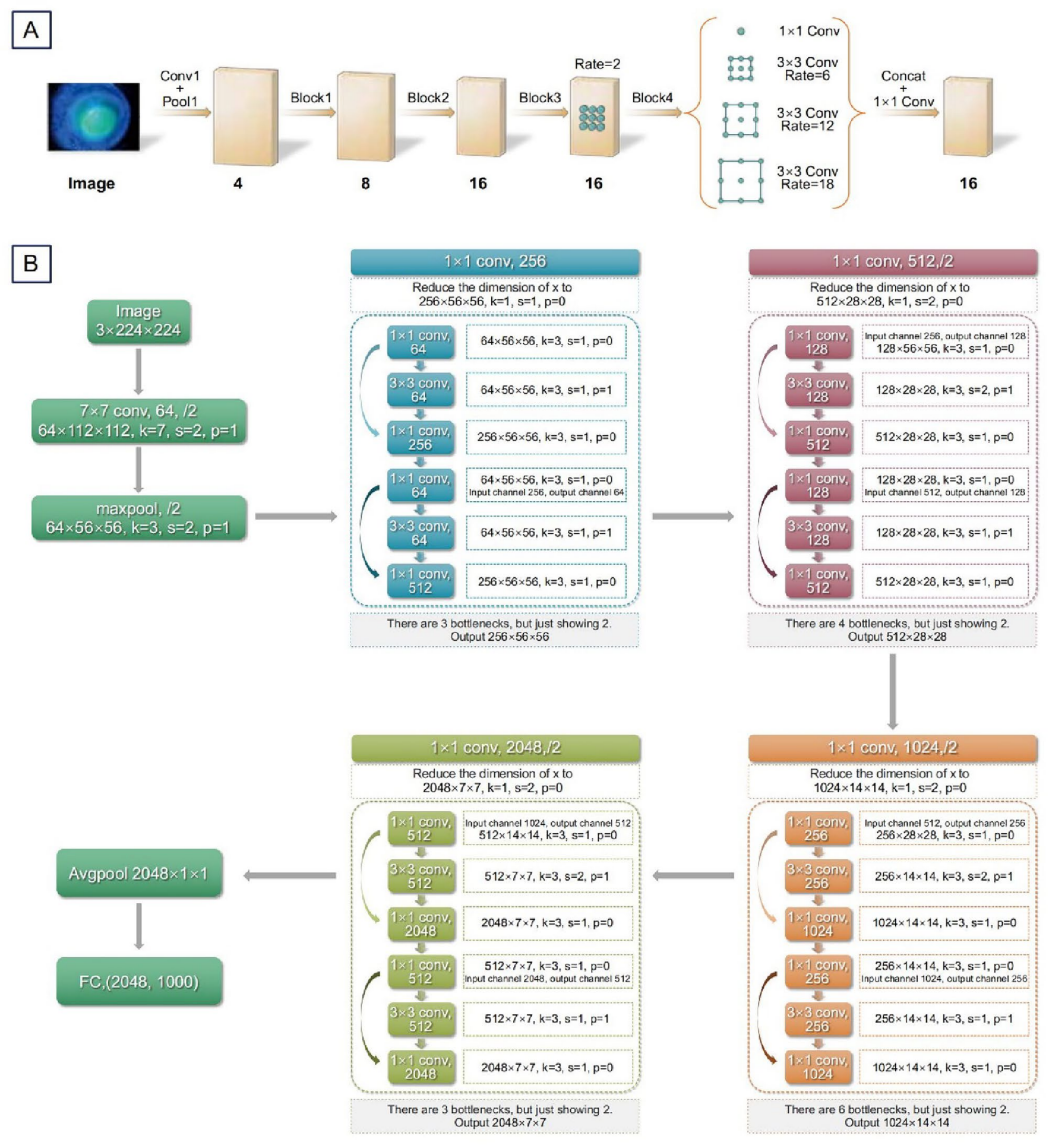
The Fig. 2A and 2B are shown ASPP in the Deeplab V3 segmentation model and Architecture of ResNet50. (**A**) illustrates the segmentation model used in our study, which is based on the DeepLabV3 architecture. (**B**) Represents the classification model for corneal scars, which is constructed based on the ResNet50 architecture.

The Deeplab series of segmentation models introduced dilated convolution technology, adding a “atrous” to the convolution operation to increase the receptive field. The ASPP^18^ network can effectively capture multiscale information at various rates.(Supplementary) DeeplabV3 also adds a branch of ASPP to improve the overall view of the image. Specifically, it initially uses GAP to compress the resolution of the feature map to 1 × 1 and then utilizes a 1 × 1 convolution to adjust the number of channels to 256. Finally, the image resolution was adjusted to the target resolution through batch normalization and bilinear interpolation upsampling. For each Region of Interest (ROI) type, we trained separate classification models. All models were fine-tuned using transfer learning algorithms, based on model parameters pre-trained on the MS COCO dataset (Fig. 2A).

### Classification model

ResNet has been widely used in various feature-extraction applications. The deeper the layer number of the deep learning network, the stronger is the expression ability. However, after the CNN network reached a certain depth and deepened further, the classification performance no longer improved, but the network convergence slowed and the accuracy decreased; thus, the classification performance and accuracy did not improve. Through the connection of residual blocks, gradient dispersion in the training process can be effectively solved. Similarly, for the classification of corneal scars, we utilized transfer learning, initializing the models with parameters pre-trained on ImageNet. We trained a ResNet-50 model with the following hyperparameters: Batch size: 32, Initial learning rate (init_lr): 0.01, with cosine learning decay the same as segmentation models, Epochs: 50, Optimizer: SGD (Fig. 2B and Supplementary).

### Feature building

Using the classification and segmentation algorithm, we performed automatic segmentation and classification of the ROI of the slit-lamp images. To obtain a better prognostic model, we must further quantify the features of the data identified by the algorithm and develop our prediction model.

〔Corneal scar〕

1. Corneal automatic classification through the classification network to obtain the label of each category and the corresponding probability.
2. Grading the percentage of the corneal scar area to the corneal area.
  $$class_{CSs} = \left\{ {\begin{array}{*{20}l} 1 \hfill & {{\text{ if 0}}{.00} \le \frac{{area_{CSs} }}{{area_{corneal} }} \le 0.25} \hfill \\ 2 \hfill & {{\text{ if 0}}{.25 < }\frac{{area_{CSs} }}{{area_{corneal} }} \le 0.50} \hfill \\ 3 \hfill & {{\text{ if 0}}{.50 < }\frac{{area_{CSs} }}{{area_{corneal} }} \le 0.75} \hfill \\ 4 \hfill & {{\text{ if 0}}{.75 < }\frac{{area_{CSs} }}{{area_{corneal} }} \le 1.00} \hfill \\ \end{array} } \right.$$
  The area_CSs_ variable in the formula is corneal scar area and the area_corneal_ variable in the formula is corneal area.
3. Whether corneal scar blocks the pupil.
  $$corneal\_{overlap}_{CSs}=\left\{\begin{array}{ll}0& \text{ if }are{a}_{CSs}\cap are{a}_{corneal}=\phi \\ 1& \text{ else}\end{array}\right.$$
4. Number of corneal quadrants occupied by the corneal scar

〔Corneal descementocele〕

1. Grading the percentage of the corneal descementocele
  $$class_{CD} = \left\{ {\begin{array}{*{20}l} 1 \hfill & {{\text{ if 0}}{.00} \le \frac{{area_{CD} }}{{area_{corneal} }} \le 0.25} \hfill \\ 2 \hfill & {{\text{ if 0}}{.25 < }\frac{{area_{CD} }}{{area_{corneal} }} \le 0.50} \hfill \\ 3 \hfill & {{\text{ if 0}}{.50 < }\frac{{area_{CD} }}{{area_{corneal} }} \le 0.75} \hfill \\ 4 \hfill & {{\text{ if 0}}{.75 < }\frac{{area_{CD} }}{{area_{corneal} }} \le 1.00} \hfill \\ \end{array} } \right.$$
  The area_CD_ variable in the formula is corneal descementocele area and the area_corneal_ variable in the formula is corneal area.

〔Anterior corneal abscess〕

1. Grading the percentage of abscess depth in the anterior chamber to the longitudinal diameter of the cornea.
  $$class_{ACA} = \left\{ {\begin{array}{*{20}l} 1 \hfill & {{\text{ if 0}}{.00} \le \frac{{area_{ACA} }}{{area_{corneal} }} \le 0.25} \hfill \\ 2 \hfill & {{\text{ if 0}}{.25 < }\frac{{area_{ACA} }}{{area_{corneal} }} \le 0.50} \hfill \\ 3 \hfill & {{\text{ if 0}}{.50 < }\frac{{area_{ACA} }}{{area_{corneal} }} \le 0.75} \hfill \\ 4 \hfill & {{\text{ if 0}}{.75 < }\frac{{area_{ACA} }}{{area_{corneal} }} \le 1.00} \hfill \\ \end{array} } \right.$$
  The area_ACA_ variable in the formula is anterior corneal abscess area and the area_corneal_ variable in the formula is corneal area.

〔Blue-light corneal ulcer〕

1. Percentage grading of the corneal ulcer area to the Blue-light corneal ulcer.
  $$class_{CU} = \left\{ {\begin{array}{*{20}l} 1 \hfill & {{\text{ if 0}}{.00} \le \frac{{area_{CU} }}{{area_{corneal} }} \le 0.25} \hfill \\ 2 \hfill & {{\text{ if 0}}{.25 < }\frac{{area_{CU} }}{{area_{corneal} }} \le 0.50} \hfill \\ 3 \hfill & {{\text{ if 0}}{.50 < }\frac{{area_{CU} }}{{area_{corneal} }} \le 0.75} \hfill \\ 4 \hfill & {{\text{ if 0}}{.75 < }\frac{{area_{CU} }}{{area_{corneal} }} \le 1.00} \hfill \\ \end{array} } \right.$$
  The area_CU_ variable in the formula is blue-light corneal ulcer area and the area_corneal_ variable in the formula is corneal area.
2. Whether corneal ulcers block pupils under blue light.
  $$corneal\_{overlap}_{CU}=\left\{\begin{array}{ll}0& \text{ if }are{a}_{CU}\cap are{a}_{corneal}=\phi \\ 1& \text{ else}\end{array}\right.$$
3. Number of corneal quadrants occupied by Blue-light corneal ulcer.

〔Corneal neovascularization〕

1. Percentage grading of the corneal neovascularization area to the corneal area.
  $$class_{CN} = \left\{ {\begin{array}{*{20}l} 1 \hfill & {{\text{ if 0}}{.00} \le \frac{{area_{CN} }}{{area_{corneal} }} \le 0.25} \hfill \\ 2 \hfill & {{\text{ if 0}}{.25 < }\frac{{area_{CN} }}{{area_{corneal} }} \le 0.50} \hfill \\ 3 \hfill & {{\text{ if 0}}{.50 < }\frac{{area_{CN} }}{{area_{corneal} }} \le 0.75} \hfill \\ 4 \hfill & {{\text{ if 0}}{.75 < }\frac{{area_{CN} }}{{area_{corneal} }} \le 1.00} \hfill \\ \end{array} } \right.$$
  The area_CN_ variable in the formula is corneal neovascularization area and the area_corneal_ variable in the formula is corneal area.
2. Whether corneal neovascularization blocks the pupil.
  $$corneal\_{overlap}_{CN}=\left\{\begin{array}{ll}0& \text{ if }are{a}_{CN}\cap are{a}_{corneal}=\phi \\ 1& \text{ else}\end{array}\right.$$
3. The number of corneal neovascularization in the corneal quadrant.

### Model construction

We converted the results automatically recognized by the artificial intelligence algorithm into features, combined them with patients’ corresponding clinical features, carried out a feature pre-fusion method, and built the final prognostic model.

Before building the model, we used the LASSO model to screen these features for different tasks and reduce the dimensions of the features to a certain extent. After filtering the features, we used common machine learning algorithms such as Gradient Boosting (XGB)^19^ and LightGBM, among other models, for algorithm verification.

## Results

### Segmentation model

We used Python 3.7 and scikit-learn v. 1.0.2 to build the model for the segmentation task of lesion identification, and DICE and IOU as evaluation indicators for the segmentation model effect.

All the tasks were trained and verified using the DeeplabV3 segmentation model. The accuracy rate, IoU, Dice, and other evaluation indicators increased steadily. For all segmentation tasks, the accuracy of the anterior corneal abscess was 96.86. The accuracy of corneal descelementocele was 91.64. The accuracy of blue-light corneal ulcer was 90.51. The accuracy of corneal neovascularization was 93.97. The accuracy of the corneal scar was 92.88. (Table 1 and Supplemental Fig. 6).

**Table 1:** The training and verification data of DeeplabV3 segmentation model.

Task	Abscess in the anterior chamber	Expansion area of the rear elastie layer	Blue light ulcer	Corneal neovascularization	Scar of cornea
global ac	96.86	91.64	90.51	93.97	92.88
mloU	83.25	61.59	69.35	77.93	77.59
mDice	90.47	73.49	81.07	87.12	86.90
Ace_background	98.63	96.13	95.32	99.02	98.45
Ace_corneal	94.41	87.89	87.98	85.96	81.57
Ace_pupil	90.21	68.37	65.93	81.48	88.87
Ace_lesions	77.96	33.55	86.11	80.63	81.07
loU_background	97.88	91.88	94.53	96.46	96.50
IoU_corneal	87.26	75.48	64.56	79.61	76.51
loU_pupil	79.94	47.98	57.25	67.06	64.15
loU_lesions	67.91	31.00	61.06	68.58	73.19
Dice_background	98.92	95.77	97.18	98.20	98.22
Dice_corneal	93.20	86.03	78.46	88.64	86.69
Dice_pupil	88.85	64.85	72.82	80.28	78.16
Dice_lesions	80.89	47.33	75.82	81.36	84.52

### Classification model

**Figure 3:**
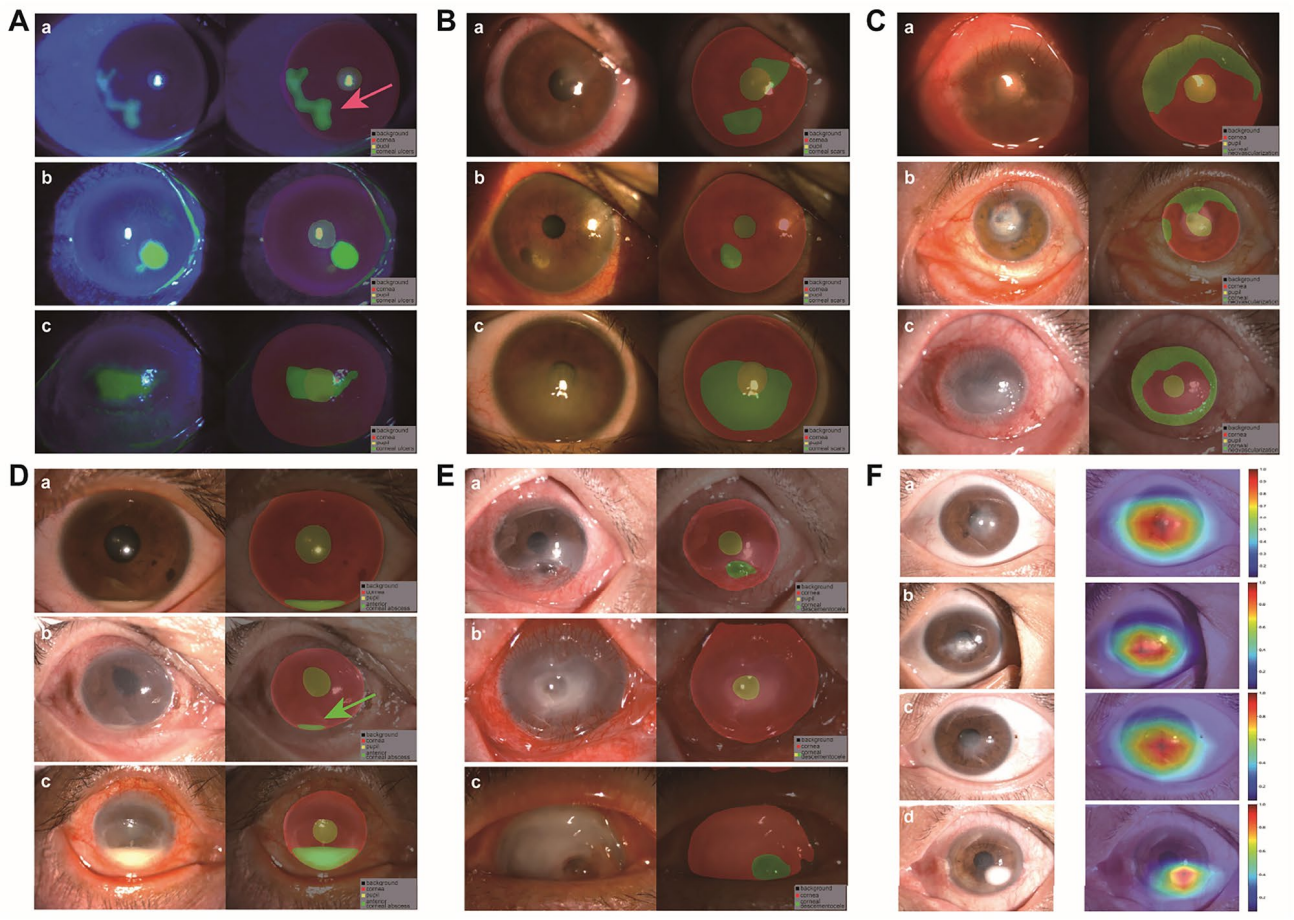
Illustrates the results of the segmentation and classification models. Figures (**A**–**E**) display the segmentation results of five types of lesions under blue light, including blue-light corneal ulcer, corneal scar, corneal neovascularisation, anterior corneal abscess and corneal descementocele. Among these, the identification of the anterior corneal abscess is the most effective. The red arrow indicates fluorescent leakage due to corneal epithelial defect, so the identification area is slightly larger than the actual situation. The green arrow indicates that the model can accurately identify the height of a small amount of pus in the anterior chamber. Figure (**F**) and Supplemental Fig. 5 show the visualisation results of the Grad-CAM classification model for corneal scars.These visualizations are generated automatically, locating regions for closer examination after a patient is seen by a consulting ophthalmologist. The bluer the color, the lower the attention of the model; the redder the color, the higher the attention of the model.

For corneal scar lesion classification, the accuracy of the final classification model was 69.76. In order to make the model’s decision-making process more transparent and investigate its interpretability, gradient-weighted class activation mapping (Grad-CAM) was applied to visualize the models. We used the gradient information of the last convolutional layer of the CNNs for weighted fusion to obtain a class activation map that high-lights the important regions of the classification target image (Fig. 3).

### Prognostic model

In the task of predicting patient outcomes after 1 month, the best model results for ulcer perforation and visual impairment were XGBoost 0.81 (0.63–1.00, 95% CI) and XGBoost 0.77 (0.63–0.91, 95% CI), respectively.

In the task of predicting patient outcomes after 3 months, the best model results for ulcer perforation and visual impairment were XGBoost 0.97 (0.92–1.00, 95% CI) and LightGBM 0.98 (0.94–1.00, 95% CI), respectively. (Table 2, Supplemental Table 2 and Supplemental Fig. 3, 7).

**Table 2:** The metrics of the segmentation model for visual impairment and corneal perforation tasks.

Model	After 1 month	After 1 month	After 1 month	After 1 month	After 3 months	After 3 months	After 3 months	After 3 months
Name	XGBoost	XGBoost	XGBoost	XGBoost	XGBoost	XGBoost	LightGBM	LightGBM
Task	Corneal perforation-train	Corneal perforation-test	Improvement of vision-train	Improvement of vision-test	Corneal perforation-train	Corneal perforation-test	Improvement of vision-train	Improvement of vision-test
Accuracy	0.97	0.85	0.95	0.68	0.97	0.91	0.97	0.97
AUC	0.99	0.81	0.99	0.77	0.99	0.97	0.99	0.98
95%CI	0.98–1.00	0.63–1.00	0.99–1.00	0.63–0.91	0.99–1.00	0.92–1.00	0.98–1.00	0.94–1.00
Sensitivity	1.00	0.71	0.97	0.62	1.00	1.00	0.96	0.90
Specificity	0.92	0.87	0.98	0.80	0.96	0.93	1.00	1.00
PPV	0.95	0.50	0.94	0.73	0.92	0.66	0.97	0.96
NPV	0.97	0.86	0.97	0.63	0.98	0.94	0.95	1.00
Precision	0.95	0.50	0.94	0.73	0.92	0.66	0.97	0.96
Recall	1.00	0.71	0.97	0.62	1.00	1.00	0.96	0.90
F1	0.97	0.58	0.95	0.67	0.96	0.80	0.97	0.93

### Model evaluation

For all segmentation tasks, the accuracy of the DeepLabV3 segmentation model is above 90%, and the highest in anterior corneal abscess is 96.86%. Gradient-weighted class activation mapping (Grad-CAM) is applied to visualise the models. The visualised results align with clinical expectations, and location recognition is accurate.

For the XGBoost model trained on the “corneal perforation” task, the AUC values are 0.99 after 1 month and 0.99 after 3 months. These values indicate excellent discrimination ability in training and test datasets, with minimal change over 3 months. In the “improvement of vision” task, the XGBoost model shows AUC values of 0.99 after 1 month and 0.99 after 3 months for the training set, indicating outstanding performance. However, the AUC values for the test set drop to 0.77 after 1 month and remain consistent at 0.97 after 3 months, suggesting a decrease in model performance when generalised to new data after 1 month, which improves significantly by the 3-months mark.

The LightGBM model performs similarly to the XGBoost model in the “improvement of vision” task, with AUC values of 0.99 after 1 month and 0.99 after 3 months for the training set. The test set AUC values are 0.98 after 1 month and 3 months, indicating excellent and consistent generalisation ability.

Overall, both XGBoost and LightGBM models exhibit strong discriminative capabilities, as reflected by the high AUC values, especially in the training sets. The performance in the test sets also generally remains high, with some fluctuations observed in the “improvement of vision” task for the XGBoost model. These results suggest that both models are effective for “corneal perforation” and “improvement of vision,” with LightGBM showing slightly better generalisation in the test set for the latter task.

**Figure 4:**
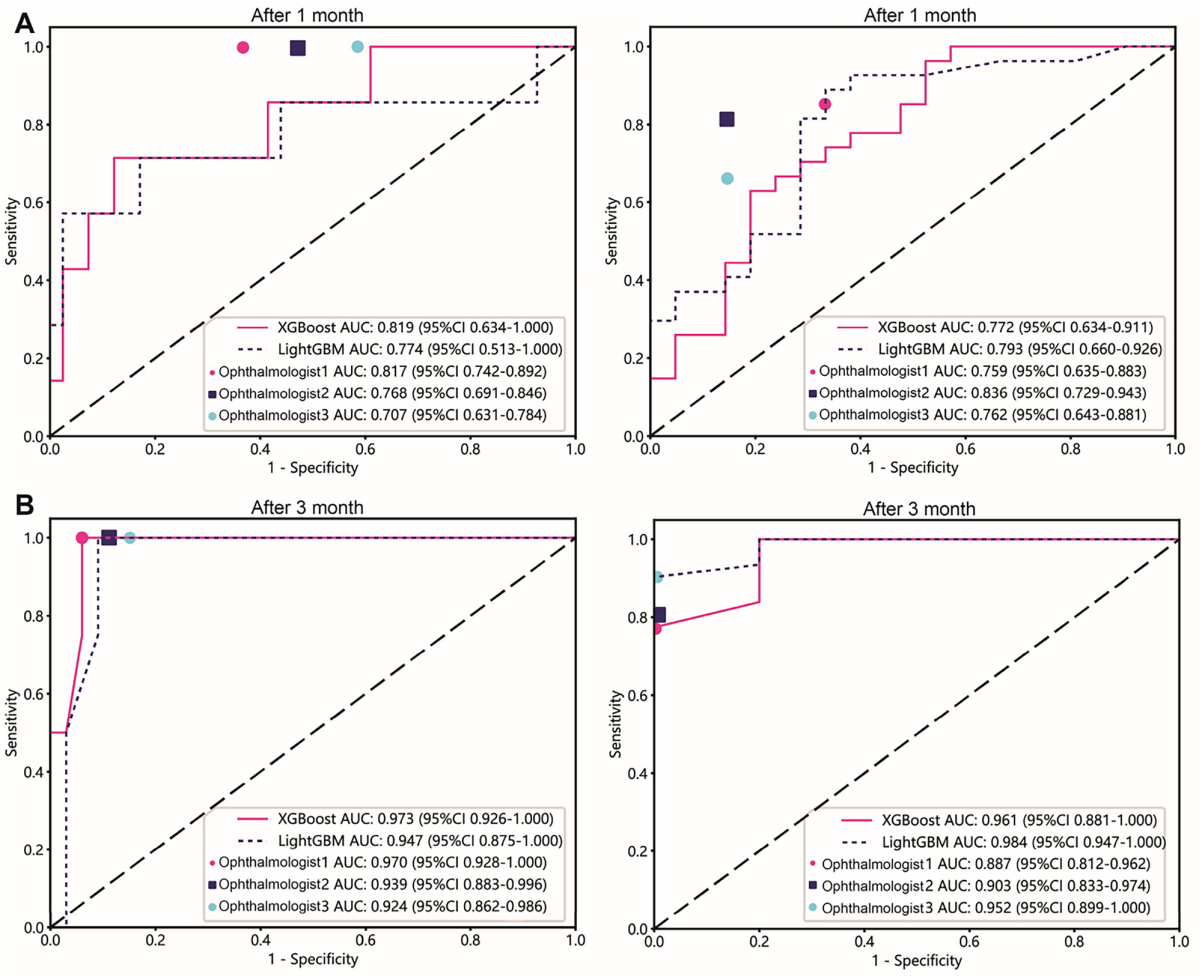
This study investigated the proposed deep learning algorithm and the ability of ophthalmologists to predict the prognosis of corneal ulcers. Three ophthalmologists were evaluated: Ophthalmologist 1, a keratopathy expert, representing senior experience, and Ophthalmologists 2 and 3, third-year residents, representing junior experience. Figure 4A shows the XGBoost model, LightGBM model and predictions by three ophthalmologists for perforated ulcer and vision improvement outcomes after 1 month. While assessing the predicted outcome of corneal perforation after 1 month, Ophthalmologist 1 predicted a slightly lower AUC value than the XGBoost model, while Ophthalmologists 2 and 3 predicted a slightly lower AUC value than the LightGBM model. For predicting visual impairment outcomes after 1 month, Ophthalmologist 2 provided greater accuracy than Ophthalmologist 1 due to the model jitter issue. Figure 4B shows the XGBoost model, the LightGBM model and predictions by three ophthalmologists for ulcer perforation and vision improvement after 3 months. The results of corneal perforation prediction after 3 months were similar to those after 1 month, with the XGBoost model outperforming the three ophthalmologists. For predicting visual impairment outcomes after 3 months, the LightGBM model achieved the highest predictive AUC value, followed by the XGBoost model, with both models performing better than the three ophthalmologists.

For the prognostic model, we fused these features with our clinical features based on the predicted results of each model using feature construction and applied the machine learning algorithm to two different aspects and corresponding tasks in 1- and 3-month intervals.(Supplemental Fig. 4) In our study, we introduced evaluations by three ophthalmologists (Ophthalmologist 1 represents senior experience; Ophthalmologists 2 and 3 represent junior experience). Additionally, we selected models for comparative analysis. We discovered that our models yield encouraging results in predicting tasks related to corneal perforation and vision improvement (Fig. 4).

## Discussion

Our study innovatively developed an automated prognostic model system that demonstrated high accuracy in predicting corneal perforation and visual impairment in patients with corneal ulcers. Given the feasibility and non-invasive advantages of anterior segment imaging, intelligent systems have great potential to facilitate the objective assessment of corneal ulcers’ poor prognosis and the development of individualised treatments. In addition, we created a website to visualise keratopathy and quantify it by grading.(Supplemental Fig. 2) This method helps clinicians record the changes of keratopathy more accurately and objectively in their daily diagnosis and treatment.

The segmentation model has obtained good results in identifying the five types of corneal ulcer lesions listed above, with the identification of an anterior chamber abscess yielding the best results. An abscess in the anterior chamber is a clear and stable white liquid plane formed by the deposition of inflammatory cells in the lower corner of the chamber. Therefore, the discrimination task is relatively simple, and the overall results are satisfactory.

With the development of AI, several models of eye diseases, such as the keratoconus prognosis model^20^, the human eye ageing prediction model^21^, and the recurrent optic neuritis prognosis model^22^, have been established in some studies. More diagnostic models based on slit-lamp images have also been designed^23,24^. In previous studies, Qasmieh et al. also used the SUSTech-SYSU public dataset to propose and compare two high-precision automatic positioning systems of corneal ulcer regions and found the deep learning method more accurate than traditional image processing technology. However, a large training dataset is required for model construction^25^. Based on the SUSTech-SYSU public dataset, our study added the original data of several hospitals to improve the accuracy of model training, which enabled the > 90% accuracy of our model in terms of identifying the four keratitis lesions.

The highlight of this study is that, firstly, we adopted a multi-centre data set to improve data reliability and model generalisation ability, so as to improve the segmentation and classification model efficacies based on deep learning. Then, the algorithmic formula was used to quantify the keratopathy, and the concept of classification was introduced to improve the fault tolerance of the initial modelling. The scoring technique involved a novel approach to the logical judgement challenge of how to assess pupil-affected keratopathy in a two-dimensional representation of three-dimensional space. Our automated prognostic model may support the decision-making of inexperienced ophthalmologists regarding the management of corneal ulcer treatment. Most importantly, this study lays the foundation for more fully automated prognostic analysis systems based on deep learning and machine learning in the future. Previous studies have shown that segmentation algorithms can accurately identify focal areas in eye images, such as inflammatory infiltration or ulcer area size^26–39^.(Supplemental Table 1) The DeeplabV3 segmentation model was used in our study. The positioning capability of this segmentation model makes the subsequent analysis more targeted. After identifying the diseased area, the segmentation model can extract its features in more detail, helping clinicians more accurately judge the nature and severity of corneal ulcer lesions. In addition, to improve the accuracy of the identification of corneal ulcer lesions, we used the segmentation algorithm and machine learning to integrate the local features and the overall features of the slit lamp image. For different types of corneal ulcers, the areas and features that may require attention vary. The segmentation algorithm and feature characterisation improve the model’s adaptability. This method not only can better identify the lesion but also provides detailed information about its size, shape, and location and whether the pupil is blocked, which helps primary physicians to make diagnoses and treatment plans for corneal ulcers.

There are some limitations to this study. Corneal photographs tend to exhibit greater complexity due to light reflection, which can affect the training quality of deep learning models due to background clutter and other issues. In addition, the accuracy of the corneal scar classification model herein was low, which may be related to intra-class variation. In the prognostic model, we used 5-step cross validation to improve its efficacy. However, the limited sample size and absence of external data sets for validation may reduce the predictive model’s accuracy. In addition, this is a retrospective study, and selection bias is inevitable in all retrospective analyses, especially given the relatively small sample size from a single centre. Therefore, further multi-centre large-scale studies are needed to verify the scalability of the prognostic model established in this study.

In this study, an automatic prognosis model system for patients with corneal ulcers was established based on deep learning and machine learning techniques. Furthermore, slit-lamp images combined with deep learning technology were used for the first time to accurately predict the outcome of corneal ulcer patients with perforation and visual impairment. Therefore, the model system can be used as an auxiliary tool for clinical analysis of the prognosis of corneal ulcers.

### Acquisition of data

This prognostic study was approved by the Ethics Committee of the First Affiliated Hospital of Guangxi Medical University. (2022-E413-01) . Written or verbal informed consent was not obtained from any participants because the Ethics Committee of the First Affiliated Hospital of Guangxi Medical University waived the need for individual informed consent, as this study had a non-interventional retrospective design and all data were analyzed anonymously. This study followed the Transparent Reporting of a Multivariable Prediction Model for Individual Prognosis or Diagnosis (TRIPOD) reporting guideline. In addition, our entire study follows the Reporting Guidelines for Artificial Intelligence in Medical Research, providing maximum assurance that the entire end-to-end pathway of the technology is reliable and repeatable when applied to similar populations^40^. All methods were performed in accordance with the relevant guidelines and regulations.

### Supplementary Information

Supplementary Figures.

Supplementary Information.

Supplementary Tables.

## Data Availability

The dataset analysed in the current study is not publicly available, but is available from the corresponding author on reasonable request.
