# Supplementary material for: Establishment of a corneal ulcer prognostic model based on machine learning
Source: Sci Rep. 2024 Jul 12;14:16154. doi: 10.1038/s41598-024-66608-7 (PMC11245505; doi:10.1038/s41598-024-66608-7)
Supplement: Supplementary file 1 — Supplementary Figures. [file 41598_2024_66608_MOESM1_ESM.doc]

**Suppl.Fig.1** shows the inclusion criteria and exclusion criteria of this prognostic model.


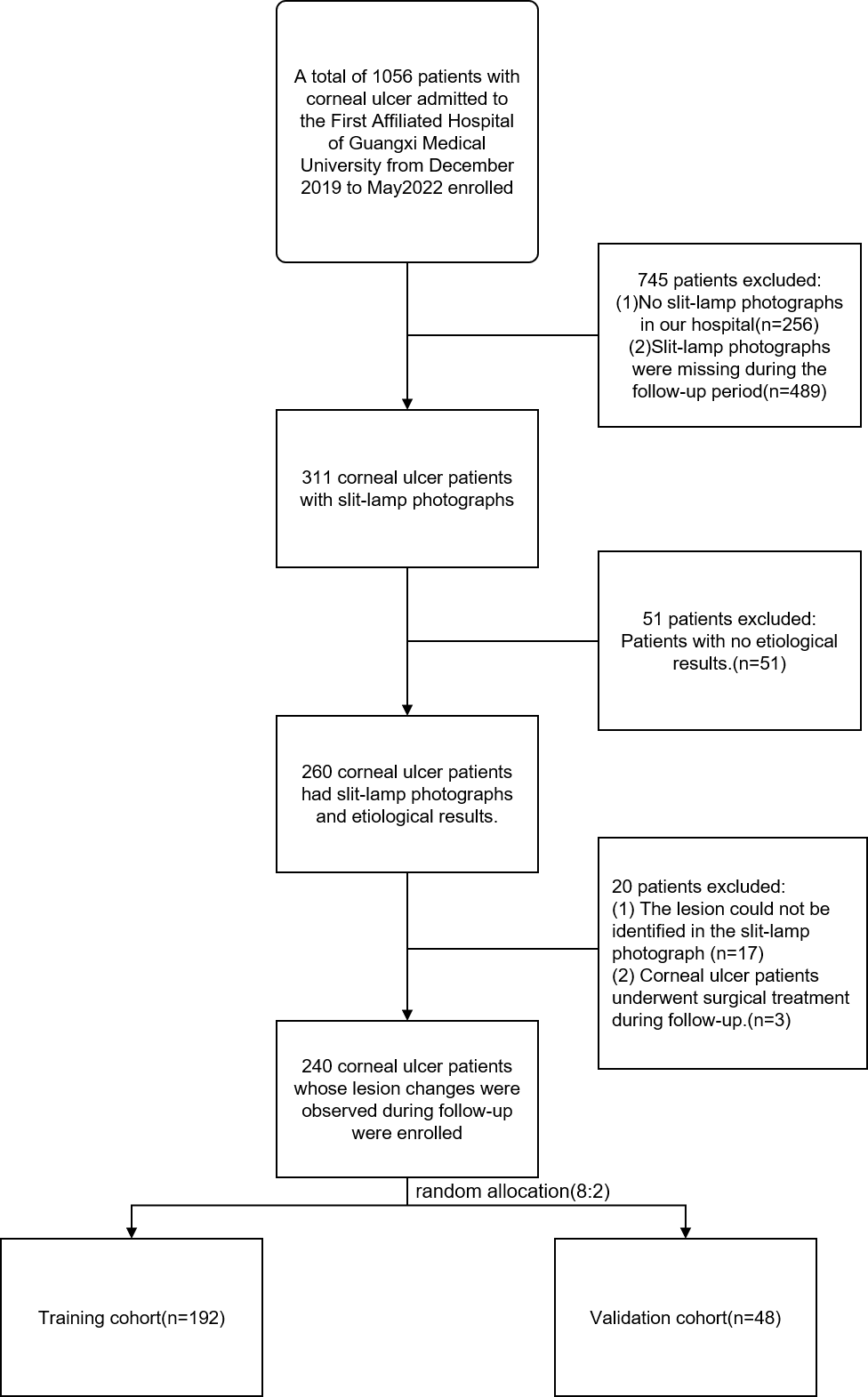


**Suppl. Fig. 2** shows a website to visualise keratopathy and quantify it by grading.


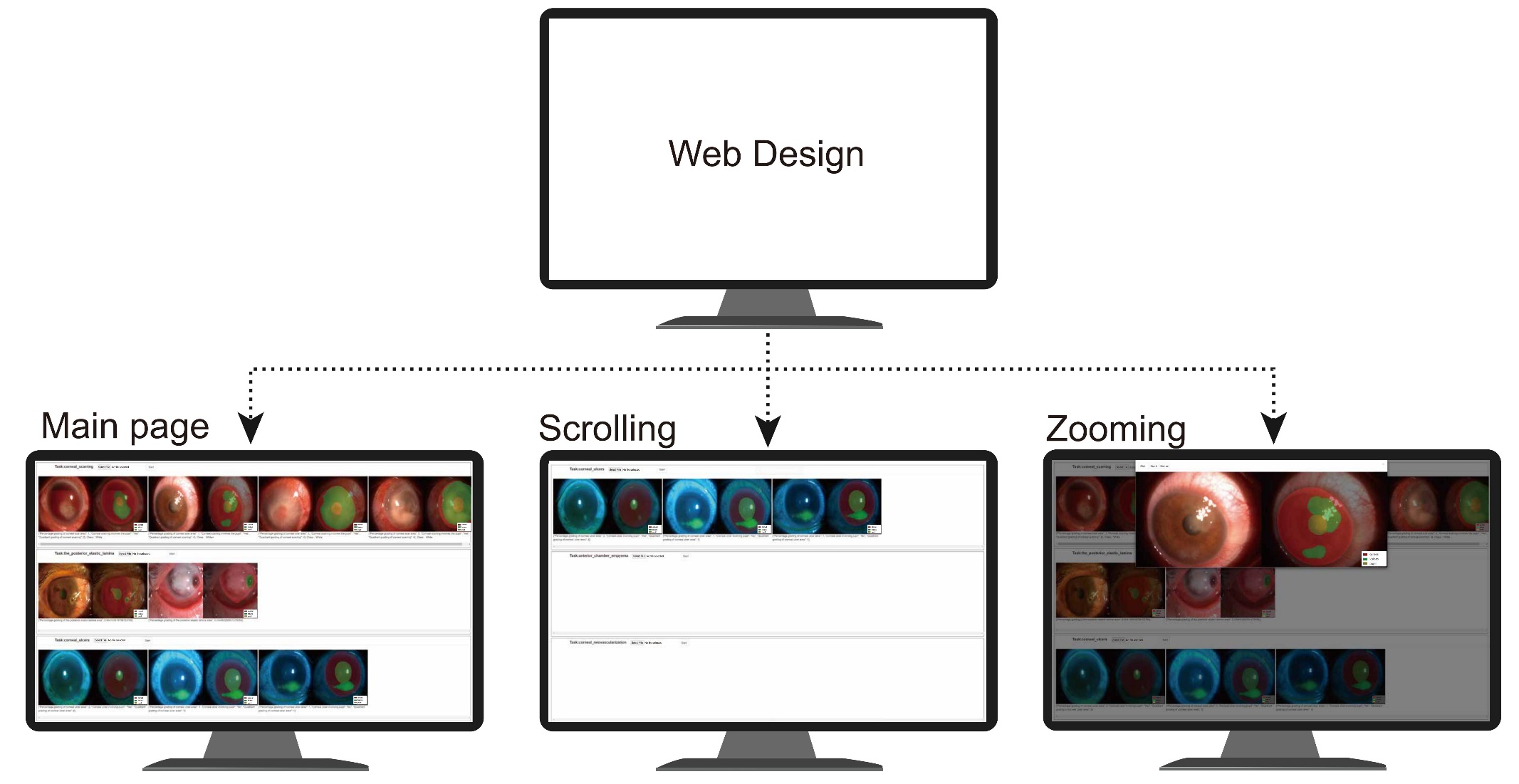


**Suppl.Fig.3** shows confusion matrix for another 4 classification.


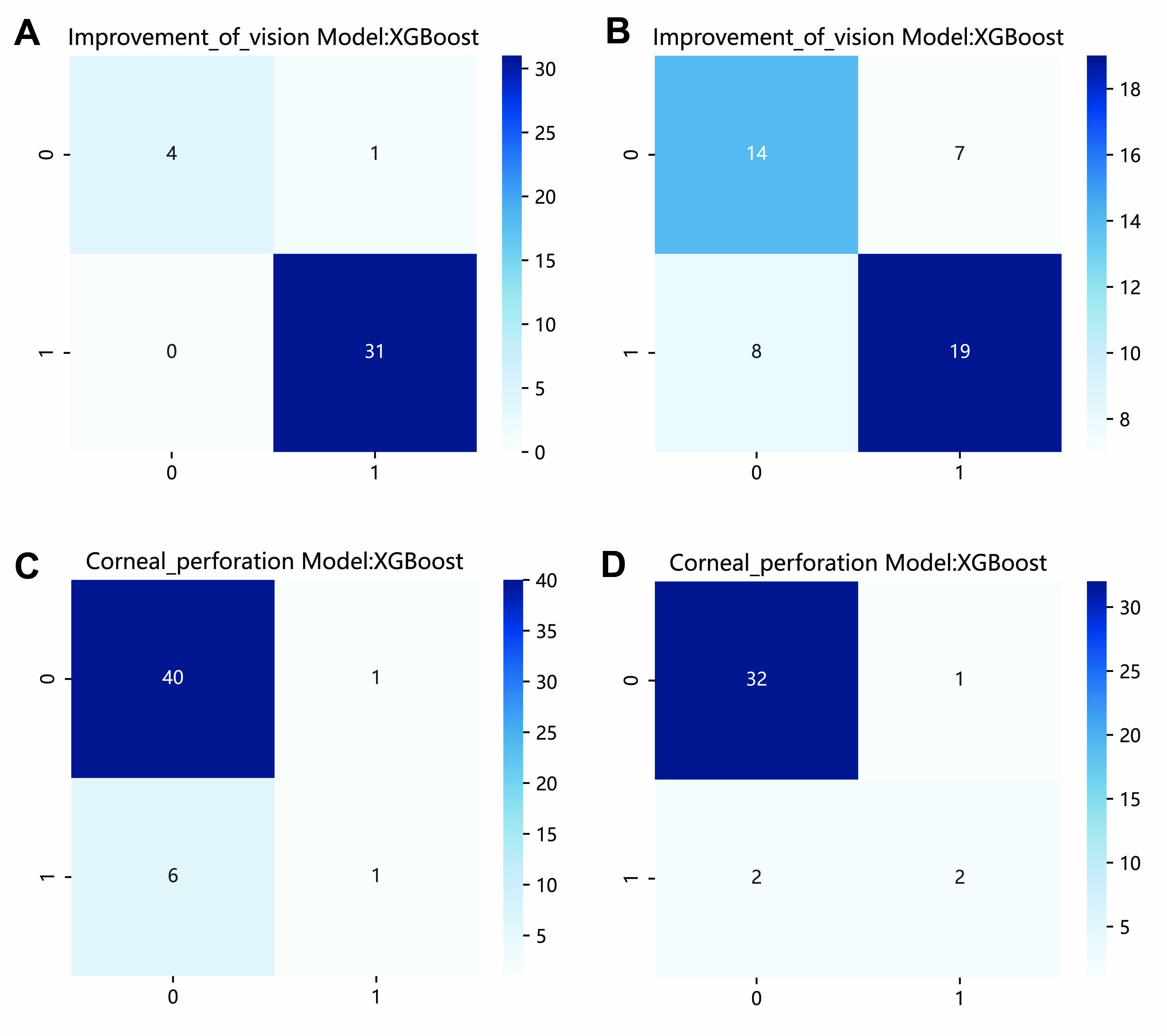


**Suppl.Fig.4**. The nomograms are used to improve the interpretability of the model results. Nomogram for predicting corneal perforation in patients with keratitis one month after onset (Figure 4A). Nomogram for predicting corneal perforation in patients with keratitis three months after onset (Figure 4B). Nomogram for predicting visual impairment in patients with keratitis one month after onset (Figure 4C). Nomogram for predicting visual impairment in patients with keratitis three months after onset (Figure 4D).


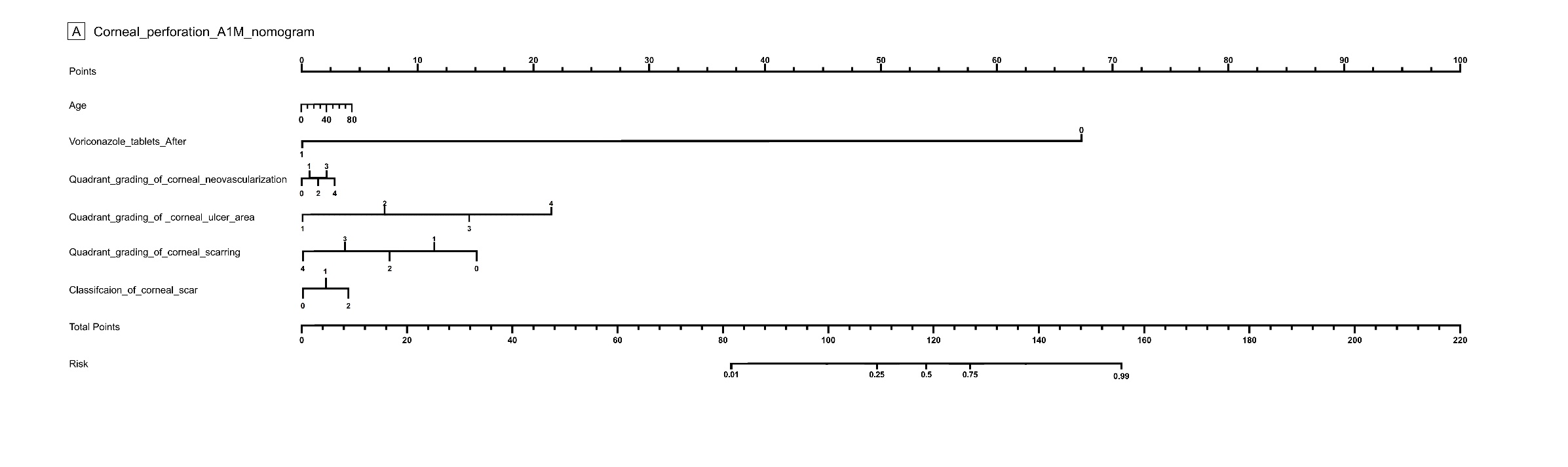


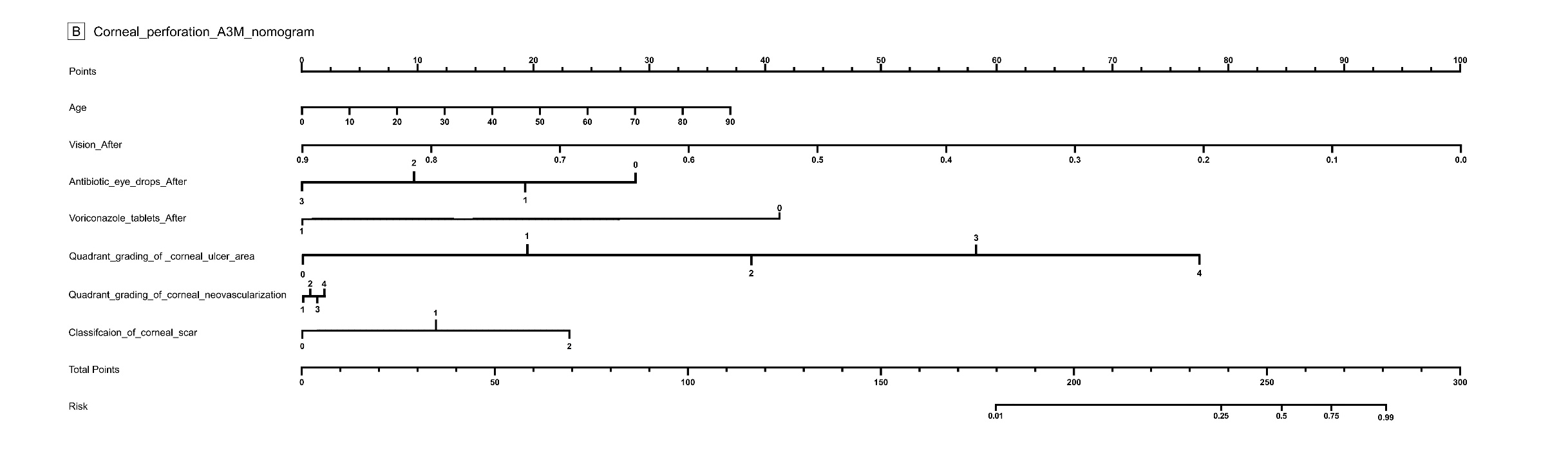


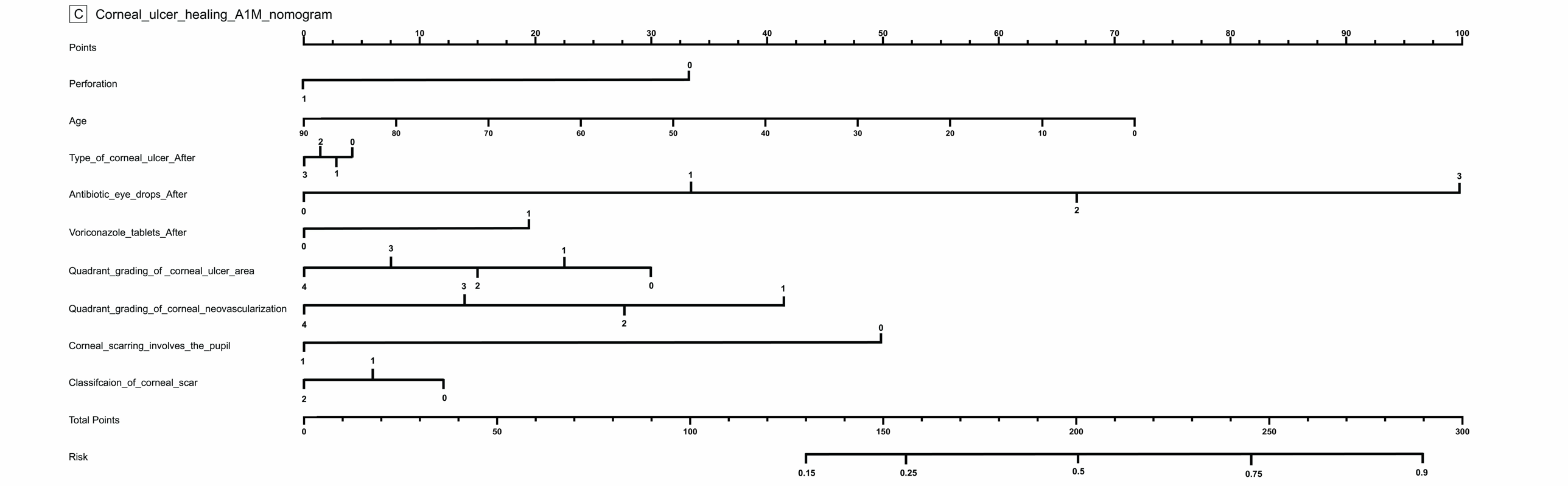


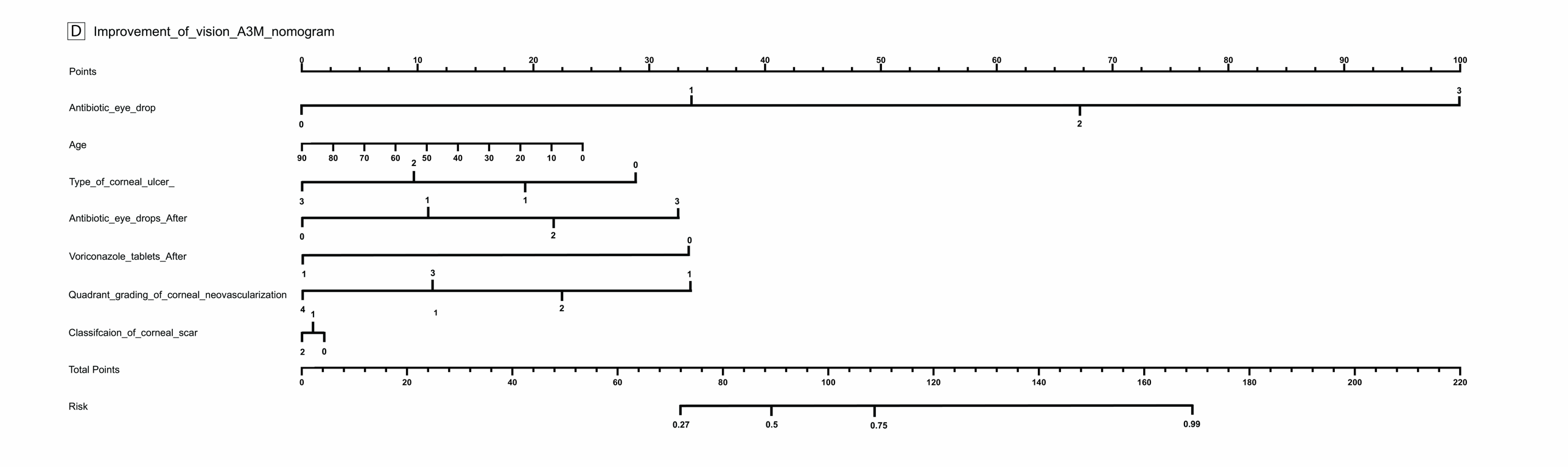


Suppl.Fig.5 are the gradient-weighted class activation mapping (Grad-CAM) was applied to visualize the models.


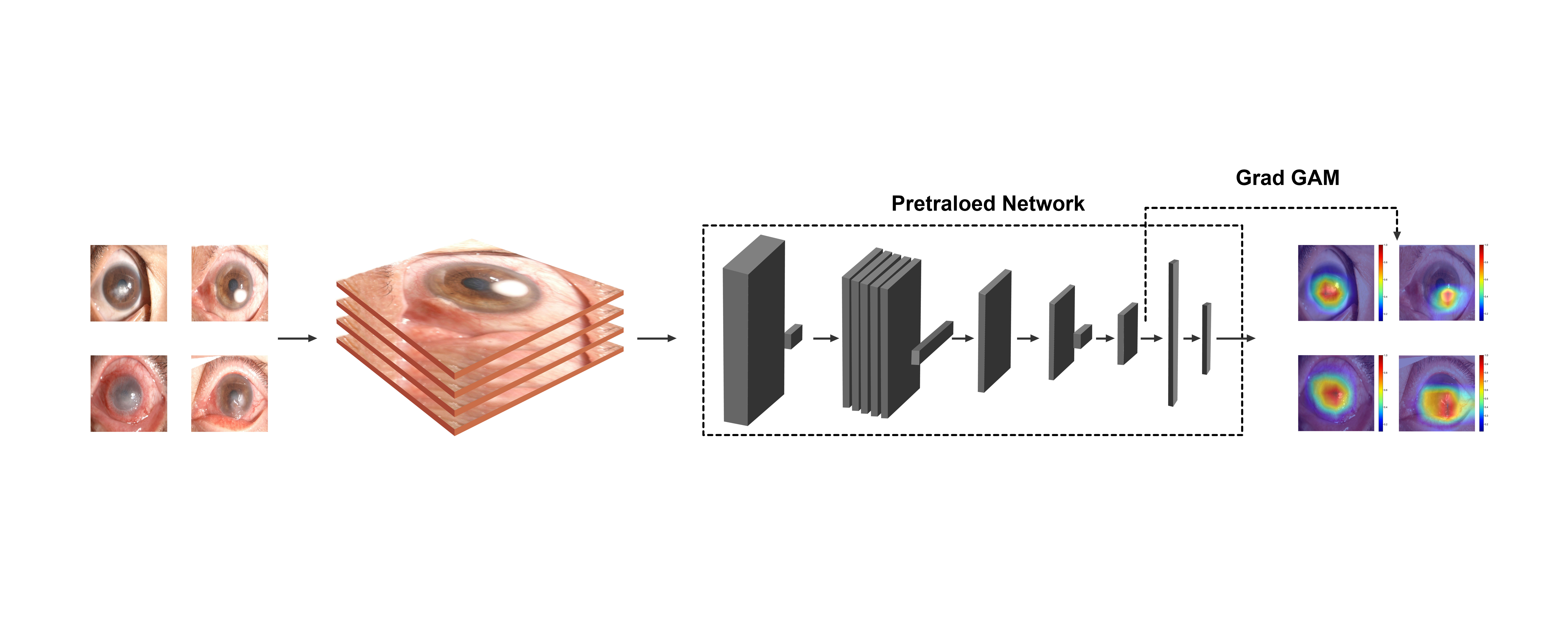


Suppl.Fig.6A-E illustrate the training process of our models for each distinct task, displaying the variation curves of Accuracy (Acc), Mean Intersection over Union (mIOU), and DICE scores.


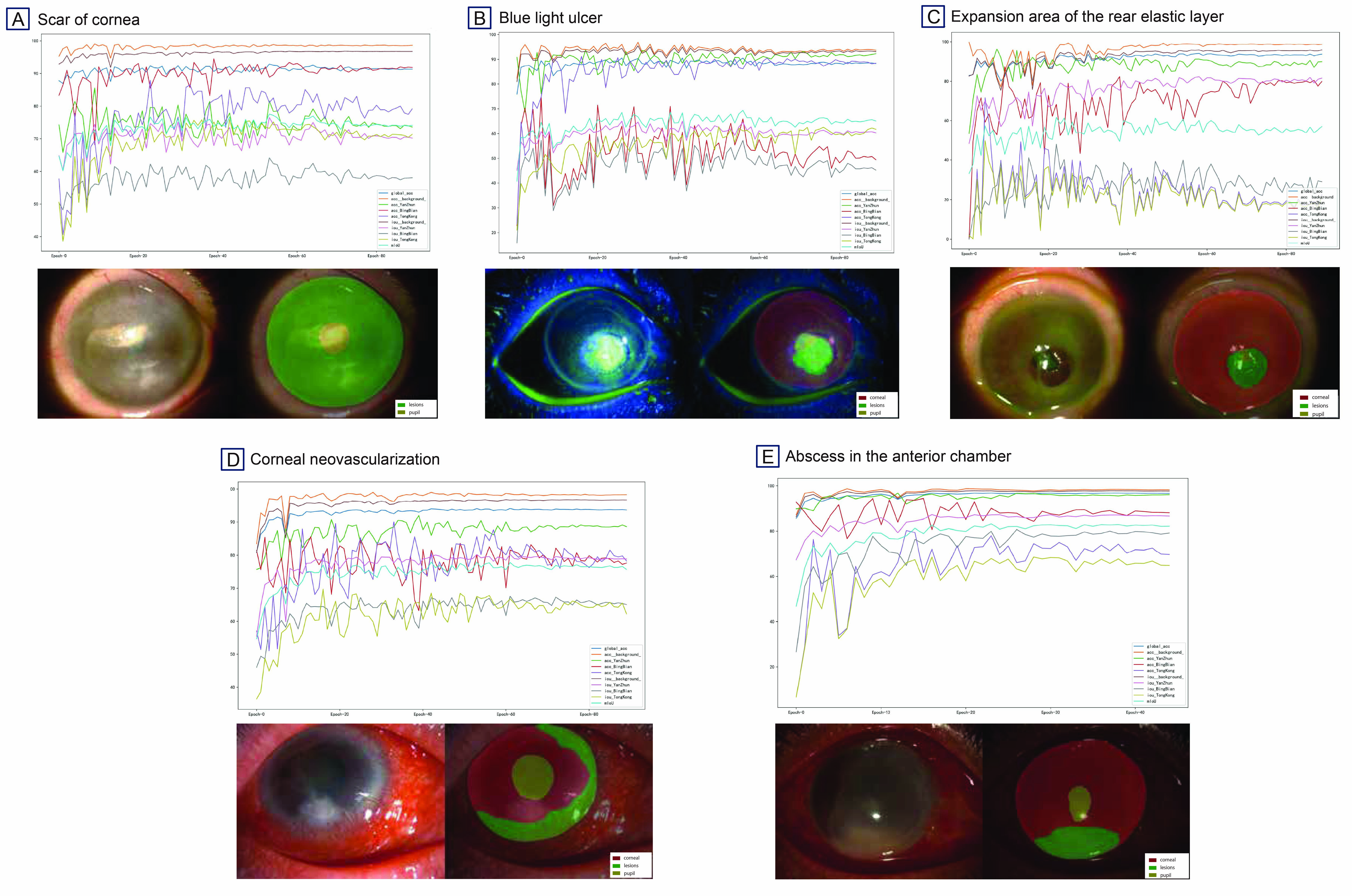


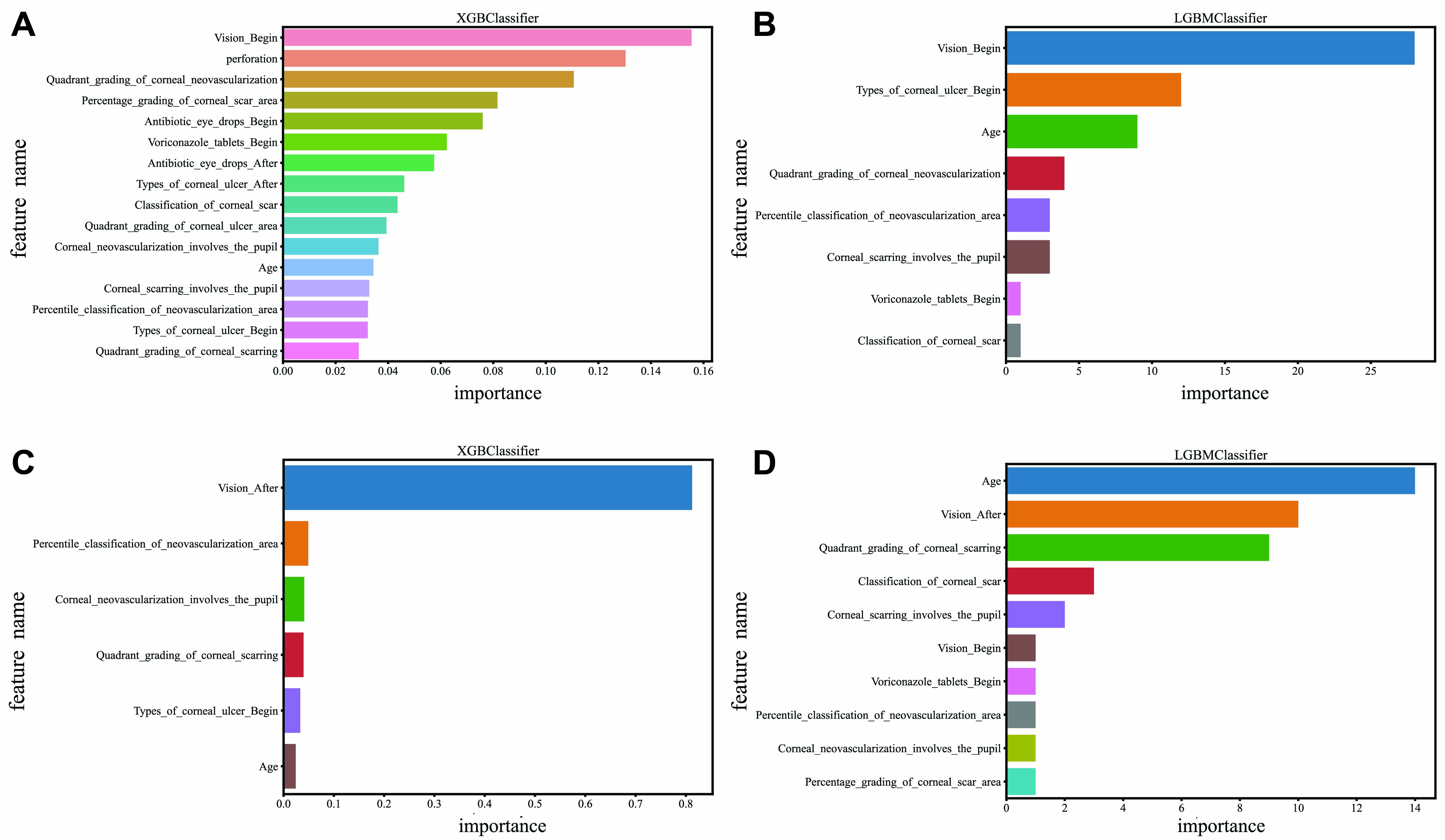
Suppl.Fig.7A-B illustrate the feature importance analysis results from XGBoost and lightGBM models 1 month after diagnosis and Fig.7C-D illustrate the feature importance analysis results from XGBoost and lightGBM models 3 months after diagnosis.
