# Supplementary material for: Establishment of a corneal ulcer prognostic model based on machine learning
Source: Sci Rep. 2024 Jul 12;14:16154. doi: 10.1038/s41598-024-66608-7 (PMC11245505; doi:10.1038/s41598-024-66608-7)
Supplement: Supplementary file 2 — Supplementary Information. [file 41598_2024_66608_MOESM2_ESM.docx]

**Technical details supplement**

The deep learning models in this study were trained using robust hardware: an Intel 13900k CPU, 64GB RAM, and an NVIDIA RTX 4090 GPU. The system operated on Windows 11. Analytical work was conducted using Python version 3.7.12 and statsmodels version 0.13.2, and machine learning model development utilized the scikit-learn version 1.0.2 interface.

Segmentation Models: For each Region of Interest (ROI) type, we trained separate classification models. All models were fine-tuned using transfer learning algorithms, based on model parameters pre-trained on the MS COCO dataset. The hyperparameters for these models are as follows: Epochs: 45，Batch size: 4，Optimizer: SGD (Stochastic Gradient Descent)

For the learning rate, we employed a carefully designed strategy. The initial learning rate was set to 0.01, and we used a cosine decay schedule, whereby the learning rate gradually decreases with the increase in the number of epochs. Specifically:

$$\eta_{t}=\eta_{min}^{i}+\frac{1}{2}\left( \eta_{max}^{i}-\eta_{min}^{i} \right)\left( 1+cos\left( \frac{T_{cur}}{T_{i}}\pi\right) \right)$$

The minimum learning rate, , is set to 0, while the maximum learning rate, , is set at 0.01. The parameter denotes the number of iteration epochs which is 45.

Classification Models for Corneal Scars: Similarly, for the classification of corneal scars, we utilized transfer learning, initializing the models with parameters pre-trained on ImageNet. We trained a ResNet-50 model with the following hyperparameters: Batch size: 32，Initial learning rate (init_lr): 0.01, with cosine learning decay the same as segmentation models，Epochs: 50, Optimizer: SGD.

**Further explanation of ASPP and atrous convolution**

The acronym ASPP stands for "Atrous Spatial Pyramid Pooling." In the context of image processing and computer vision, particularly within the DeepLab series of networks, ASPP is a crucial component designed to enhance the model's ability to process features at different scales.

ASPP plays a significant role in capturing multi-scale information within images by employing atrous convolution, also known as dilated convolution. This type of convolution uses kernels with different dilation rates, which effectively increase the receptive field—the area of the input image that the model can observe—without adding extra computational costs. The design of the ASPP module was inspired by Spatial Pyramid Pooling (SPP), which has proven effective in resampling features at different scales for image classification tasks.

Specifically, the ASPP module comprises several parallel atrous convolution layers, each with a distinct dilation rate, allowing it to capture features at various scales. These multi-scale features are then combined to form a rich, multi-scale feature representation, which is essential for accurately delineating boundaries in semantic segmentation tasks.

In summary, ASPP is an important module in the DeepLab model that improves semantic segmentation performance by integrating multi-scale information, thereby enhancing the model's ability to recognize objects within images.
