# Supplementary material for: Establishment of a corneal ulcer prognostic model based on machine learning
Source: Sci Rep. 2024 Jul 12;14:16154. doi: 10.1038/s41598-024-66608-7 (PMC11245505; doi:10.1038/s41598-024-66608-7)
Supplement: Supplementary file 3 — Supplementary Tables. [file 41598_2024_66608_MOESM3_ESM.doc]

**Suppl.Tab 1.**Summary table of deep learning models in infectious keratitis. Key: AUROC = area under the receiver operating curve,BK=bacteria keratitis,FK=fungal keratitis,IK=infectious keratitis,HSK = herpes simplex keratitis,AK = acanthamoeba keratitis,MK=Microbial keratitis,VK=viral keratitis.

| **Authors** | **Year** | **Research object** | **Sample Type** | **Sample Size** | **Sample Source** | **AI Algorithm** | **The Accuracy of the primary Algorithm(95%CI)** | **The ROC of the primary Algorithm(95% CI)** |
| --- | --- | --- | --- | --- | --- | --- | --- | --- |
| Hung  et al. | 2021 | Culture-proven IK | Slit lamp pictures | 1330 slit-  lamp  photo  graphs | two medical centers in Taiwan | RN50,  RN101,  DN121,  DN 161,  DN 169,  DN201,  IC-v3,  ENB3 | the deep learning algorithm achieved the highest average accuracy of 80.0%. | RN50 = 0.82  RN101 = 0.77  DN121 = 0.82  DN161 = 0.85  DN 169 = 0.78  DN201 = 0.80  IC-v3 = 0.82  ENB3 = 0.75 |
| Kuo  et al. | 2021 | Culture-proven IK or three specialists have a consensus impression of one type of IK | Slit lamp pictures | 1512 slit-  lamp  photo  graphs | five referral centers | RN50,  RNXt50,  SE-RN50,  DN121,  ENB0,  ENB1,  ENB2,  ENB3, | The diagnostic accuracy of these models (ranged from 69 to 72%) is comparable to that of the ophthalmologist (66% to 74%). | RN50 = 0.75  RNXt50 = 0.74  SE-RN50 = 0.75  DN121 = 0.75  ENB0 = 0.73  ENB1 = 0.75  ENB2 = 0.74  ENB3 = 0.76 |
| Ghosh  et al. | 2022 | Culture  proven  IK | Slit- lamp pictures | 2167 anterior segment images |  | VGG19,  DenseNet121,  RestNet50 | _ | _ |
| Hu et al. | 2023 | Culture  proven  IK | Slit- lamp pictures | 2757 slit- lamp images | Single center | VGG16,  ResNet34,  InceptionV4,  DenseNet121,  ViT-Base,  EffecientNetV2-M | The best classification algorithm was EffecientNetV2-M, with an accuracy of 0.735. | _ |
| Koyama  et al. | 2021 | Culture  proven  IK | Slit- lamp pictures | 4306 slit- lamp images | — | RN-50,  IC,  RN-v2 | AK = 97.9  BK = 90.7  FK = 95  HSK = 92.3 | AK = 0.995 (0.991–0.998)  BK = 0.963 (0.952–0.973)  FK = 0.975 (0.964–0.984)  HSK = 0.946 (0.926–0.964) |
| Zhang et al. | 2022 | Culture  proven  IK | Slit- lamp pictures | 4830 slit -lamp images | — | ResNet18, ResNet50,18 DenseNet121, DenseNet169,19 EfficientNet-b0, EfficientNet-b5, EfficientNet-b7,20 ResNext101_32x8d, and ResNext101_32x16d | BK=70.27%  FK=77.71%  AK=83.81%  HSK=79.31% | BK=0.86  FK=0.91  AK=0.96  HSK=0.98 |
| Kuo et al. | 2020 | clinically suspected MK | Slit -lamp pictures | 288 slit -lamp images | _ | DenseNet | _ | _ |
| Kuo et al. | 2022 | BK | Slit -lamp pictures | 929 slit -lamp images | Chang Gung Research Database | ResNet50、DenseNet121、ResNeXt50、SE-ResNet50、EfficientNets B0 ~ B3 | The best ensemble 4-DL model showed the highest accuracy (72.1%) among the ensemble models. | - |
| Won YK | 2023 | BK and FK | Slit -lamp pictures | 684 anterior segment photographs | SMC dataset and Open source dataset | Baseline (ResNet-50)、Baseline + LGM、Baseline + MAM、  Baseline+ MAM+LGM | — | — |
| Redd TK | 2022 | BK and FK | handheld cameras collected from patients | ImageNet imaging database | 4 centers in South India. | MobileNetV2,  DenseNet201，ResNet152V2，Xception,VGG19 | — | MobileNet model=0.83 |
| Natarajan R | 2022 | VK | Slit- lamp pictures | 307 diffusely illuminated Slit lamp photographs | Single center | DenseNet-201，  AlexNet,,VGGNet, ResNet, Inception | DenseNet=72% | — |
| Loo J | 2021 | MK | Slit l-amp pictures | 133 Slit -lamp pictures | Two centers | nn-UNet | _ | _ |
| Li Z | 2021 | Keratitis,other cornea abnormalities, and normal cornea | Slit- lamp pictures | 6,567 slit-lamp images. | Five centers | DenseNet121,  Inception-v3,  ResNet50 | _ | DenseNet121=0.968 (95% CI, 0.958-0.977) |
| Kogachi K | 2023 | infectious keratitis | handheld cameras | 1970 images | Single center | DenseNet,  MobileNet | _ | DenseNet=0.48(95%CI, 0.40-0.57)  MobileNet=0.52 (95% CI, 0.44–0.60) |

**Suppl. Tab. 2** shows the clinical baseline table.

| Feature_name | ALL | test | train | P value |
| --- | --- | --- | --- | --- |
| Age | 54.39±15.69 | 55.03±14.03 | 54.23±16.12 | 0.78358699 |
| BCVA | 0.17±0.22 | 0.12±0.17 | 0.18±0.23 | 0.13878874 |
| Height_of_hypopyon | 1.14±0.07 | 1.13±0.04 | 1.15±0.07 | 0.25049606 |
| Percentage_grading_of_the_posterior_elastic_lamina_area | 0.13±0.03 | 0.12±0.04 | 0.13±0.02 | 0.689994097 |
| Percentile_classification_of_ncovascularization_area | 1.08±0.12 | 1.1±0.22 | 1.07±0.08 | 0.039879722 |
| Corneal_neovascularization _involves_the_pupil | 0.37±0.22 | 0.43±0.28 | 0.35±0.20 | 0.042403532 |
| Quadrant_grading_of_corneal_neovascularization | 2.87±0.47 | 3.01±0.48 | 2.83±0.46 | 0.036951463 |
| Percentage grading_of_coneal_sear_area | 1.40±0.59 | 1.39±0.56 | 1.40±0.60 | 0.95075729 |
| Corneal _scarring_involves_the pupil | 0.86±0.27 | 0.87±0.27 | 0.86±0.27 | 0.925203073 |
| Quadrant_grading_of_corneal_scarring | 2.79±1.01 | 2.81±1.07 | 2.79±1.00 | 0.883184856 |
| Classification_of_corneal_scar | 1.39±0.64 | 1.26±0.70 | 1.42±0.62 | 0.149895559 |
| Types_of_comeal_ulcer |  |  |  | 0.361747172 |
| 0 | 56(30.43) | 9(24.32) | 47(31.97) |  |
| 1 | 72(39.13) | 14( 37.84) | 58(39.46) |  |
| 2 | 28(15.22) | 5(13.51) | 23(15.65) |  |
| 3 | 28(15.22) | 9(24.32) | 19(12.93) |  |
| perforation |  |  |  | 0.085648791 |
| 0 | 56(30.43) | 34(91.89) | 137(93.20) |  |
| 1 | 72(39.13) | 3(8.11) | 10(6.80) |  |
| Types of comeal_ulcer |  |  |  | 0.233250135 |
| 0 | 58(31.52) | 9(24.32) | 49133.33) |  |
| 1 | 72(39.13) | 14(37.84) | 58(39.46) |  |
| 2 | 28(15.22) | 5(13.51) | 23(15.65) |  |
| 3 | 26(14.13) | 9(24.32) | 17(11.56) |  |
| Corneal uleer healing |  |  |  | 0.027249276 |
| 0 | 61(33.15) | 13(35.14) | 48(32.65) |  |
| 1 | 123(66.85) | 24(64.86) | 99(67.35) |  |
| Coneal perforation |  |  |  | 0.045124635 |
| 0 | 165(89.67) | 33(89.19) | 132(89.80) |  |
| 1 | 19(10.33) | 4(10.81) | 15(10.20) |  |
| Visual impairment |  |  |  | 0.029043099 |
| 0 | 27(14.67) | 4( 10.81) | 23(15.65) |  |
| 1 | 157(85.33) | 33(89.19) | 124(84.35) |  |
| Percentage_grading_of_corneal_ulcer_area |  |  |  | 0.607847645 |
| 0 | 170(92.39) | 36(97.30) | 134(91.16) |  |
| 1 | 9(4.89) | 1(2.70) | 8(5.44) |  |
| 2 | 4(2.17) | null | 4(2.72) |  |
| 3 | 1(0.54) | null | 1(0.68) |  |
| Corneal_ulcer_involving_pupil |  |  |  | 0.382864717 |
| 0 | 7(3.80) | null | 7(4.76) |  |
| 1 | 177(96.20) | 37(100.00) | 140(95.24) |  |
| Quadrant_grading_of_corneal_ulcer_area |  |  |  | 0.047538035 |
| 0 | 7(3.80) | 1(2.70) | 6(4.08) |  |
| 1 | 5(2.72) | 1(2.70) | 4(2.72) |  |
| 2 | 16(8.70) | 7(18.92) | 9(6.12) |  |
| 3 | 18(9.78) | 1(2.70) | 17(11.56) |  |
| 4 | 138(75.00) | 27(72.97) | 11(75.51) |  |
